# Polymerized Selenium Nanoparticles for Folate-Receptor-Targeted Delivery of Anti-Luc-siRNA: Potential for Gene Silencing

**DOI:** 10.3390/biomedicines8040076

**Published:** 2020-04-05

**Authors:** Fiona Maiyo, Moganavelli Singh

**Affiliations:** Nano-Gene and Drug Delivery Group, Discipline of Biochemistry, University of KwaZulu-Natal, Private Bag X54001, Durban, South Africa; fimaiyo@gmail.com

**Keywords:** selenium nanoparticles, gene silencing, chitosan, folate, siRNA

## Abstract

The development of a biocompatible and nontoxic gene delivery vehicle remains a challenging task. Selenium nanoparticles (SeNPs) have the potential to increase delivery efficiency, to reduce side effects, and to improve therapeutic outcomes. In this study, chitosan (Ch) functionalized folate (FA)-targeted SeNPs were synthesized, characterized, and evaluated for their potential to bind, protect, and safely deliver F*luc*-siRNA in vitro. SeNPs of less than 100 nm were successfully synthesised and further confirmed using UV-vis and Fourier transform infrared spectroscopy, transmission electron microscopy, and nanoparticle tracking analysis. Cell viability studies were conducted in vitro in selected cancer and non-cancer cell lines. Folate receptor (FOLR1) targeted and nontargeted luciferase gene silencing studies were assessed in the transformed Hela-tat-*Luc* cell line expressing the luciferase gene. Targeted and nontargeted SeNP nanocomplexes showed minimal toxicity in all cell lines at selected *w*/*w* ratios. Maximum gene silencing was achieved at optimum *w*/*w* ratios for both nanocomplexes, with Selenium-chitosan-folic acid (SeChFA) nanocomplexes showing slightly better transgene silencing, as supported by results from docking studies showing that SeChFA nanocomplexes interacted strongly with the folate receptor (FOLR1) with high binding energy of −4.4 kcal mol^−1^.

## 1. Introduction

Cancer is a complex manifestation of abnormalities in signalling pathways that enable the cell to evade the body’s homeostatic systems. The occurrence of developmental and degenerative disorders, including cancer, mostly involve abnormal gene regulatory mechanisms. Combination therapy has been utilised as a promising strategy to address challenges faced by cancer treatment such as drug insolubility, drug resistance, and off-target toxicities, among others [1]. Gene therapy approaches in cancer therapy have shown great potential in addressing challenges faced by conventional chemotherapy with enhanced antitumour activity. It presents a highly specific and efficient form of treatment compared to other therapeutic techniques, and for this, it has gained much interest [2]. Through gene therapy, therapeutic proteins have been introduced to tumour cells that have aided in its death, such as those encoded by genes responsible for apoptosis-inducing proteins TRAIL, P53, and TNF-α [3]. The completion of human genome sequencing in 2003 facilitated the study and identification of therapeutic gene targets and have further enabled scrutiny of oncogenic genes such as those regulating apoptosis, cell cycle, cell signalling, and cell proliferation [4].

RNA interference is an evolutionarily conserved phenomenon for sequence-specific gene silencing in plants, worms, yeast, and mammals discovered at the beginning of the 21st century. It evolved as a cellular defence against viral infection and other exogenous genes such as transposons and is now considered a regulatory mechanism for controlling gene expression [4,5,6]. In gene therapy, RNAi is a novel strategy employing the use of short double-stranded RNA molecules to achieve posttranscriptional gene silencing [7]. Long double-stranded RNA molecules are cleaved in the cytoplasm into short fragments of 21–23 nucleotides by dicer to produce siRNA which is incorporated into the RISC (RNA induced silencing complex)-inducing sequence-specific silencing through RNase degradation on a target region of endogenous mRNA [4]. Small RNA duplexes that have successfully been used in RNAi include miRNA, siRNA, and shRNA [6]. However, siRNA remains the most efficient in posttranscriptional gene silencing because of its 100% complementarity to target mRNA and the ability of the guide strand to cleave mRNA repeatedly.

Moreover, siRNA has an added advantage over other drugs in terms of safety due to reduced risk of insertional mutagenesis [8]. Using RNAi technology, proteins implicated in aberrant cell cycle examples of which include kinesin spindle protein (KSP) and (PLK1) involved in carcinogenesis have been targeted for posttranscriptional silencing [9]. Currently, there are more than 20 RNAi-based therapies at clinical trials for the treatment of different cancers. TKM-PLK1, a lipid nanoparticle-based siRNA drug targeting polo-like kinase overexpressed in cancer is at clinical trials for the treatment of gastrointestinal (GI) tumours and adrenocortical carcinoma [10]. CALAA-01 is a targeted siRNA therapy which binds to transferrin on the cell surface, releasing siRNA-targeting RRM (ribonucleotide reductase M2-subunit) involved in DNA replication and has just completed phase 1 clinical trials [8,11].

Despite the signs of progress made in designing and developing siRNA targets to various oncogenes in cancer therapy, successful application has been hampered by the lack of safe and efficient delivery vehicles [12], with certain characteristics of siRNA presenting delivery challenges. Firstly, siRNA has a large molecular weight and is polyanionic in nature, making it a challenge to cross the cell membrane unaided. In addition, in vivo, siRNA has a short half-life in plasma and is rapidly degraded and cleared by the kidneys [10]. Successful delivery of siRNA therapies will require complexing to delivery vectors to facilitate transport to target cells. Moving forward, significant considerations in the success of RNAi technology are the toxicity of both delivery vector and complexes, and transfection efficacy. Successful clinical application of siRNA-mediated knockdown will ultimately depend on effective conjugation and on safe and efficient delivery of a functional nucleic acid [5].

Nanoparticles hold great promise in mediating delivery through increasing uptake, enhancing dosage, and enabling tumour targeting, thus accelerating research seen in this area in the past few years [13]. Nanoparticles are considered superior to other delivery vehicles owing to their inert nature, making them non-immunogenic as immunogenicity has been one of the greater challenges of gene delivery. Through the enhanced permeability and retention (EPR) effect, nanoparticles can successfully selectively target cancer cells; moreover, they can be modified to incorporate targeting ligands to target cancer cell receptors [14]. Inorganic nanoparticles are versatile delivery platforms and have been successfully employed in siRNA delivery. Magnetic iron oxide and gold nanoparticles have shown up to 90% gene knockdown efficiency in a Hela cell line and are currently at different stages of clinical trials for various cancers. Cellular uptake involves interaction with the membrane and transport through different pathways, and receptor-mediated endocytosis is projected to increase accumulation in tumours while minimising off-target toxicities [15]. Proper encapsulation is vital in ensuring safe and efficient transport of nucleic acid into the cytoplasm. Cationic polymers can condense siRNA to nanosized complexes capable of crossing the membrane through endocytosis. A strong interaction is desirable in maintaining stability, but after endosomal escape, siRNA should be free to interact with the RISC complex. Ionic interaction is seen to be the simplest and most effective strategy in complexing nucleic acid. Amines have mostly been used in these interactions as they maintain a positive charge at physiological pH. Chitosan is a nontoxic, naturally occurring polymer that is biocompatible, and biodegradable [16]. Protonated chitosan binds and neutralises negatively charged siRNA, compacting it; facilitates entry into the cell; and protects from degradation. Folic acid is stable, inexpensive, and non-immunogenic compared to its protein counterparts and binds to the folate receptor (FOLR1) with high affinity. Previous studies report up to 90% siRNA uptake resulting in 80% target mRNA reduction using folate-targeted siRNA in prostate and lung cancer [17].

Selenium is an essential trace element with excellent antioxidant and chemopreventive activity and is the subject of numerous reviews [18,19,20,21]. Selenium nanoparticles (SeNPs) have emerged as potential drug carriers and has been used as an essential strategy in overcoming multi-drug resistance(MDR) through a combination of gene and drug therapeutics [22,23]. Selenium conjugated to folate has been used for targeted delivery in hepatocellular carcinoma [24]. Selenium used as SeNPs have been reported to lower systemic toxicities commonly associated with traditional chemotherapeutic drugs and to synergistically assist in the improvement of the therapeutic index [24]. Furthermore, they also have been shown to exhibit increased bioavailability and biocompatibility combined with low toxicity, making them potentially favourable drug or gene carriers. This bioactivity coupled with a reported selectivity to cancer cells suggest that SeNPs may be promising targeted delivery vehicles with low side effects [25,26]. Furthermore, their biocompatibility and low cytotoxicity have rivalled other inorganic drug delivery agents. However, a significant drawback in using SeNPs in delivery has been poor uptake. In this study, we have encapsulated SeNPs in chitosan and attached folic acid as a targeting ligand to improve on these shortcomings. Furthermore, molecular docking studies were carried out to investigate the effect SeNP has on the ligand–receptor interactions of the folate receptor.

## 2. Experimental

### 2.1. Materials

Sodium selenite, ascorbic acid, chitosan (214 kDa, deacetylation degree >75%), folic acid, N, N’-dicyclohexylcarbodiimide (DCCI), deuterated dimethylsulfoxide (DMSO), MTT (3-(4,5-dimethyldiazol-2-yl)-2,5-diphenyltetrazolium bromide), and Bicinchoninic acid assay kit were purchased from Sigma Aldrich, St. Louis, MO, USA. 2-(4-(2-Hydroxyethyl)-1-piperazinyl) ethane sulphonic acid (HEPES), sodium dodecyl sulphate (SDS), Tris(hydroxymethyl-aminomethane hydrochloride (Tris-HCL), ethylenediaminetetraacetic acid (EDTA), ethidium bromide, and acridine orange were sourced from Merck, Darmstadt, Germany. Ultrapure^TM^ Agarose was supplied by Gibco Invitrogen, USA. SiGENOME non-targeting siRNA (D-001210-01) and anti-Luc siRNA (D-002050-01) were obtained from Thermo Scientific Dharmacon Products (Lafayette, CO, USA). All other chemicals used were of analytical grade with Ultrapure Milli-Q water (18 Mohm) used in all experiments. Transformed human cervical cancer cells (Hela-tat-*Luc*) stably expressing the luciferase gene was provided by the Department of Physiology, University of KwaZulu-Natal, South Africa; the human embryonic kidney cells (HEK293) were obtained from the Antiviral Gene Therapy Unit, Medical School, University of Witwatersrand; and the colorectal carcinoma (Caco-2) cells and colorectal adenocarcinoma (HT-29) cells were purchased from the American Type Culture Collection (ATCC, Manassas, VA, USA). Cells were maintained in Eagles minimum essential medium (EMEM) with L-Glutamine, supplemented with 10% fetal bovine serum (Hyclone, UT, USA) and antibiotics (100 U/mL penicillin, 100 µg/mL streptomycin) (Lonza Bio Whittaker, Verviers, Belgium).

### 2.2. Synthesis of SeNPs, SeCh, and SeChFA

Selenium nanoparticles (SeNPs) were prepared by ascorbic acid reduction, as previously described by References [27,28]. Briefly, 5 mM sodium selenite was added to 20 mM ascorbic acid with continued stirring for an h until a deep red colour was observed. The mixture was reconstituted to a final concentration of 1 mM. This was left to stir at room temperature for 1 h. Thereafter, the mixture was dialysed in a 12 kDa molecular weight cut off (MWCO) dialysis tubing against Milli-Q water for 48 hs and stored at 4 °C for further study.

Chitosan functionalisation was using one-pot synthesis. Ten ml of 0.1% chitosan (>75% deacetylated) in 1% acetic acid was added dropwise to 7.5 mL of 0.23 M ascorbic acid. This was stirred with slight heating for 30 min, after which 0.25 mL of 0.51 M aqueous sodium selenite was added to the mixture under magnetic stirring and the volume was brought up to 50 mL. Colour change from colourless to red indicated the successful formation of selenium nanoparticles, and this was left to stir for 2 h at room temperature. The mixture was dialysed in a 12-kDa MWCO dialysis tubing against 2 L Milli-Q water for 24 h to get rid of excess reactants.

Folate targeting was achieved through carbodiimide conjugation of folic acid to chitosan as has been previously documented [26] with slight modification. Forty mg of folic acid and 100 mg of DCCI was added in 15 mL of anhydrous DMSO and left to stir for 1 h at room temperature until completely dissolved. Afterwards, the activated folic acid was added dropwise to 20 mL of 0.1% chitosan under magnetic stirring and left to stir for 24 h. The mixture was coagulated by adjusting the pH to 9.0 using 0.1 M NaOH, and the coagulation was centrifuged at 2500 rpm for 10 min to remove excess solvent. This was then dialysed against Milli-Q water for 48 h. SeChFA was obtained by dissolving 1 mg/mL chitosan-folic acid (ChFA) in 0.5% acetic acid and by adding the mixture with stirring dropwise to the prepared SeNPs [29,30].

### 2.3. Nanocomplex Formation

Nanocomplexes were prepared by adding 0.5 µg of siRNA to varying amounts of prepared nanoparticles to obtain different binding ratios in a total volume of 10 µL in HBS (HEPES buffered saline (HEPES 20 mM, NaCl 150 mM) at pH 7.5. This mixture was incubated at room temperature for 1 h before use.

### 2.4. Nanoparticle and Nanocomplex Characterisation

Nanoparticle tracking analysis (NTA) (NanoSight NS500, Malvern Instruments, Worcestershire, UK) was used to study the size, zeta potential, and particle size distribution. Measurements were conducted at 25 °C and 24 V using diluted samples (Milli-Q water) to a concentration of ≈10^8^ particles/mL. Data were obtained using NanoSight NTA version 3.0 software. Morphology of nanoparticles and nanocomplexes (10 µL suspension dried on copper grids) were assessed using transmission electron microscopy (TEM, JEOL 1010 JEM, Tokyo, Japan) operating at 100 kV. Micrographs were obtained and analysed using ITEM Soft Imaging System.

Further characterization was conducted by UV-vis spectroscopy (JASCO-V-730-BIO spectrometer, JASCO Corporation, Hachioji, Japan) in the wavelength range of 190–800 nm. The degree of folic acid substitution on chitosan was determined by UV-vis spectroscopy as previously reported (Houng, 2009). FTIR spectra were obtained on a PerkinElmer Spectrum 100 FTIR spectrometer with a universal attenuated total reflection (ATR) sampling accessory in the range of 380–4000 cm^−1^.

### 2.5. siRNA Binding Studies

The ability of nanoparticles to bind siRNA was evaluated by agarose gel electrophoresis, using a 2% agarose gel containing 3 µL (1 µg/mL) ethidium bromide. Nanocomplexes at different *w*/*w* ratios were prepared as in Section 2.3, to which 3 µL gel loading dye (sucrose 40% and bromophenol 0.5%) was added before electrophoresis. Samples were loaded onto the gel and electrophoresis was conducted for 1 h at 55 V in a BioRad Mini-Sub apparatus (Richmond, CA, USA). Gels were viewed and photographed using a Vacutec SynGene (Hamburg, Germany) UV-transilluminator gel imaging system.

Ethidium bromide dye displacement assay further examined the compactness of the nanocomplexes. Ethidium bromide (0.2 µg) in 100 µL HBS was used to set the zero fluorescence. Thereafter, 0.5 µg of siRNA was added and fluorescence was set as 100%. The nanoparticles were added incrementally (1 µL) to this mixture, and fluorescence was measured using a Glomax™ Multidetection system (Promega Biosystems, Sunnyvale, CA, USA) at an excitation wavelength of 520 nm and an emission wavelength of 600 nm until a plateau in fluorescence readings was reached.

### 2.6. siRNA Protection Study

Protection of siRNA from RNase A degradation was studied using agarose gel electrophoresis. SeCh-siRNA and SeChFA-siRNA nanocomplexes at different *w*/*w* ratios obtained from gel retardation studies were incubated with 1 µL RNase A at 37 °C for 2 h. A positive control (siRNA only) and negative control (siRNA treated with RNase A) were included. Following incubation, the samples were treated with EDTA (10 mM) to inactivate the RNase A and 1 SDS (0.5% *w*/*v*) to dissociate the siRNA from the nanocomplex. After that, the samples were incubated at 55 °C for 20 min, followed by electrophoresis as in 2.5.

### 2.7. MTT Cell Viability Assay

The influence of the nanocomplexes on cell viability was determined using the MTT (3-(4,5-dimethylthiazol-2-yl)-2,5-diphenyltetrazolium bromide) assay [31]. Cells were seeded at a density of 1.7 × 10^5^ cells per well into a 48-well plate containing 200 µL of medium and were allowed to attach overnight at 37 °C in 5% CO_2_. Thereafter, the medium was replenished, nanocomplexes were added in triplicate, and cells were incubated for 48 h at 37 °C. Cells without complexes served as a control.

Thereafter, the old medium was discarded, and fresh medium containing 20 µL of MTT (5 mg/mL in phosphate buffered saline, PBS) was added to each well. The cells were incubated for 4 h at 37 °C, followed by removal of medium-MTT and addition of 200 µL of DMSO to dissolve the formazan salt. The colour change was measured on a Mindray 96A (Vacutec, Hamburg, Germany) microplate reader at 570 nm. Microsoft Excel 2016 **™** was used to generate graphs and to calculate the percentage cell viability.

### 2.8. Luciferase Gene Silencing In Vitro

Hela-tat-*Luc* cells expressing the luciferase gene were seeded at a density of 3.0 × 10^5^ cells per well in a 48-well plate containing EMEM. Cells were incubated at 37 °C for 24 h, followed by replenishment of the medium and addition of nanocomplexes at selected ratios (*w*/*w*) to the cells in triplicate. Cells were then incubated in a humidified atmosphere for 48 h at 37 °C in 5% CO_2_.

Thereafter, gene knockdown was assessed using the luciferase reporter gene assay. The medium was removed from the wells, and cells were washed twice with 100 µL PBS. Reporter cell lysis buffer (Promega Corporation, Madison, WI, USA) (80 µL) was then added to each well, and the plate was gently shaken for 15 min. The cells were scraped from the surface of the wells, and lysates were centrifuged at 12,000× *g* for 5 s to remove cell debris. Thereafter, 20 µL of the supernatant was transferred to a white 96-well plate, into which 100 µL of luciferase assay reagent (Promega Corporation, Madison, WI, USA) was directly injected, and luminescence was read on a Glomax™ Multidetection system (Promega Biosystems, Sunnyvale, CA, USA) at an absorbance of 562 nm. Total protein was determined by the standard bicinchoninic acidBCA assay using 50 µL of cell lysate. Results were represented as relative light units (RLUs) per mg protein and converted to percentage luciferase expressed against the cell only control.

Uptake through receptor-mediated endocytosis was confirmed for SeChFA nanocomplexes using a competition assay. Approximately, 50 mM folic acid was added as an excess to the cells and incubated 30 min prior to addition of nanocomplexes. Nanocomplexes were then added, and the cells were incubated for 48 h. Thereafter, the cells were subjected to the luciferase assay, as described earlier.

### 2.9. Cellular Uptake study

Selenium uptake into the cells was determined as previously reported by the authors [28]. Selenium concentration in the cells was determined by inductively coupled plasma-optical emission spectroscopy(ICP-OES) on a Perkin Elmer OES Optima 5300 DV. Briefly, the selenium concentration was noted before transfection, and following the 48 h transfection as in Section 2.8, the cell lysates were diluted to 15 mL with Milli-Q water and selenium concentration determined by ICP-OES. The selenium concentration was measured against a standard curve calibration curve using 0.2 ppm to 25 ppm of the standard stock solution. The selenium concentration is hence reported as pre-transfection and post-transfection.

### 2.10. Molecular Docking Studies

SeChFA compounds were constructed, and energy minimized using MMFF94S force-field in Avogadro software [32]. The crystal structure of the folate receptor (Protein data bank (PDB) ID: 4KM6) was obtained from the RCSB PDB protein structure [33] as the starting coordinates. Water and ions were deleted from the structure using the Chimera molecular modelling tool [34]. Compounds were docked into the in silico identified active site pockets as proposed using the Metapocket (http://projects.biotec.tu-dresden.de/metapocket/). AutoDock tools graphical interface was used to define the grid box for the selected active site pocket. A Lamarckian genetic algorithm was used to conduct docking calculations [35] in AutoDock Vina [36], with ten runs performed for the docking experiment. The docking complexes with the highest binding affinity were considered.

### 2.11. Statistical Analysis

Experiments were conducted in triplicate, and data were represented as means ± SD. Statistical analyses were done using two-way ANOVA on GraphPad Prism Version 5.04 (GraphPad Software Inc., USA), followed by Bonferroni posttests, which analysed the differences between the means. *p*-value < 0.05 was considered significant.

## 3. Results

### 3.1. Synthesis and Characterisation of Nanoparticles and Nanocomplexes

**Figure 1:**
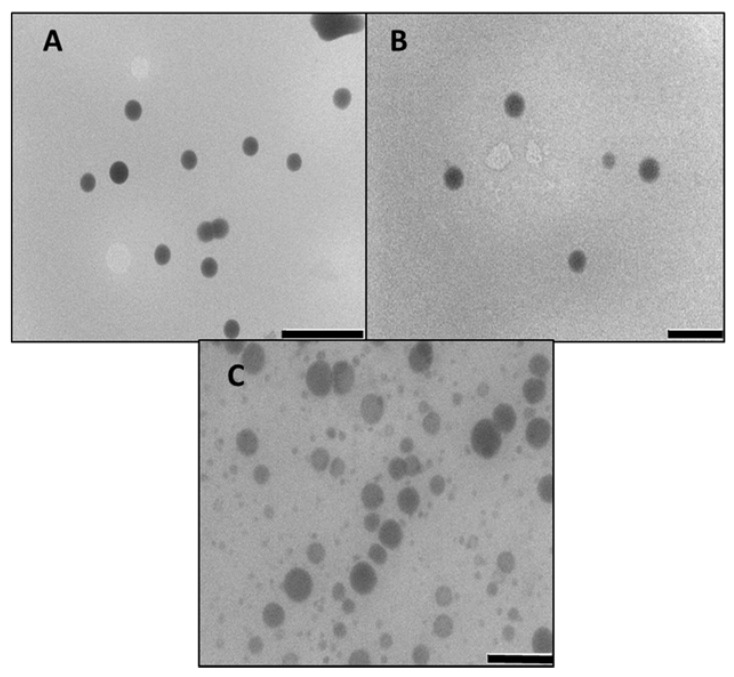
TEM images of (**A**) Selenium nanoparticles (SeNPs), (**B**) SeCh, and (**C**) SeChFA. Bar = 200 nm.

SeNPs were synthesised through reduction of sodium selenite with ascorbic acid and stabilised with chitosan. A colour change from colourless to red signified successful reduction of the selenium salt to elemental selenium. TEM images of the SeNPs presented spherical and dispersed nanoparticles, which retained their spherical morphology upon chitosan functionalization, with SeChNPs appearing homogenous and monodispersed (Figure 1).

NTA revealed SeNPs with an average diameter of 85.3 nm, which reduced to 59.6 nm and 75.6 nm upon chitosan and chitosan-folic acid conjugation, respectively (Table 1). SeCh and SeChFA nanocomplexes had slightly reduced diameters of 63.3 nm and 57.1 nm, respectively, compared to uncomplexed nanoparticles. SeNPs possessed a zeta (ζ) potential of −14.8 mV, which shifted to 21 mV after chitosan stabilisation (Table 1). Addition of the targeting folic acid moiety to the nanoparticle system led to a lower ζ potential, slightly reducing colloidal stability due to shielding of NH^+3^ groups as well as substitution with folic acid. However, upon nanocomplex formation with siRNA, the ζ potentials increased, affording relatively stable nanocomplexes (Table 1), with a good potential for gene delivery.

**Figure 2:**
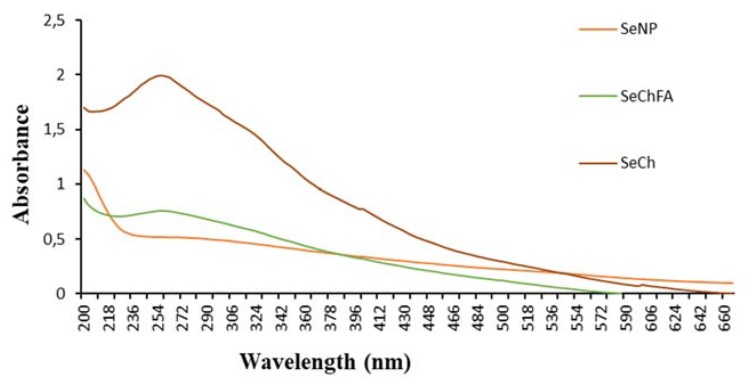
UV-vis spectroscopy of SeNPs before and after functionalisation.

UV-vis studies showed Se absorbing at below 200 nm, which is in line with studies reporting the absorption maxima of Se to be between 190–300 nm [37,38,39]. This shifted to 263 nm upon chitosan functionalisation absorbing at a higher wavelength. Addition of folic acid for targeting to the nanoparticle system saw a reduction in peak intensity to 263 nm, suggesting a slight increase in size and some agglomeration (Figure 2).

FTIR further confirmed successful stabilisation and functionalisation of SeNPs. The chitosan spectrum showed characteristic stretches at 3423 cm^−1^, 2866 cm^−1^, 1619 cm^−1^, 1151 cm^−1^, and 1078 cm^−1^ attributed to OH, CH, and NH bend O bridges and to CO. After folic acid attachment, peaks consistent with folate groups appeared 1438 cm^−1^ and 1627 cm^−1^ for C=C stretching in the aromatic ring of folic acid. These were absent in unmodified chitosan. Peaks at 1538 cm^−1^, 2850 cm^−1^, and 2927 cm^-1^ were assigned to amide I; amide II (N–H bending) was observed at 1577 cm^−1^; and a weak amide I (C=O stretching) was observed at 1655 cm^−1^ (Supplementary Information, Figures S1 and S2). This was in agreement with published literature [40].

### 3.2. Nanoparticle: siRNA Interactions

**Figure 3:**
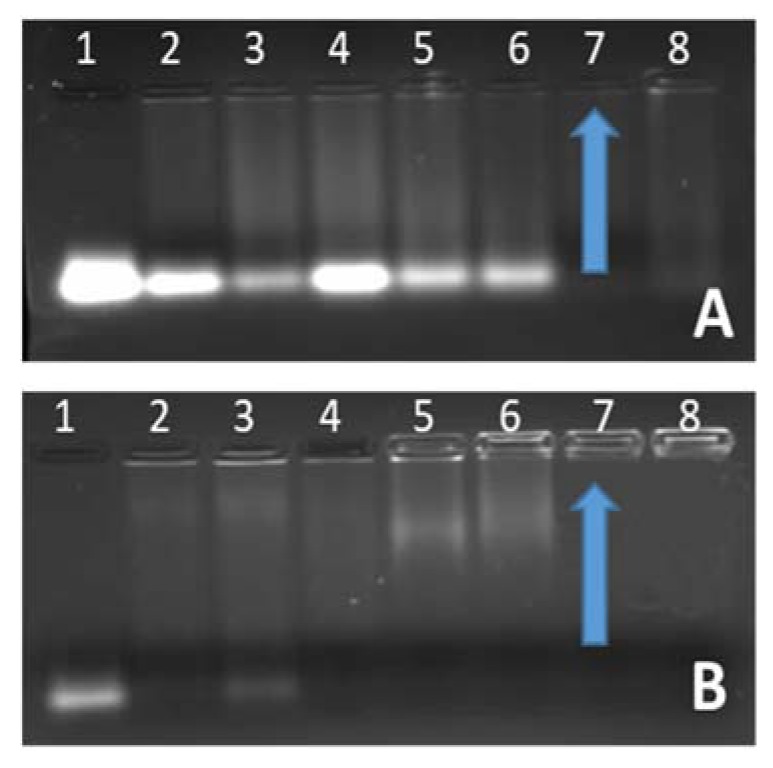
Agarose gel electrophoresis showing complexing of siRNA to nanoparticles: (**A**) SeCh and (**B**) SeChFA. Lane 1: siRNA (0.5 µg), Lanes 2–8: 0.5 µg siRNA and varying amounts of NP (**A**) SeCh (0.5, 1, 1.5, 2, 2.5, 3, and 3.5); (**B**) SeChFA (0.4, 0.5, 1, 2, 3, 4, and 5). Arrows indicate end-point/optimum binding *w*/*w* ratios.

The binding capacity of siRNA was investigated by adding varying amounts of NP to a constant amount of siRNA. siRNA migration was completely inhibited at optimum binding (*w*/*w*) ratios, which differed in different nanoparticles (Figure 3). Ratios before and after the optimum binding ratio were selected for further investigation to ascertain gene delivery at different ratios. Targeted SeChFA bound siRNA at higher ratios due to reduced positive charge and charge shielding by the conjugated folate. Furthermore, the low charge density on siRNA and its rigid structure made it difficult to form complexes. The addition of the ligand further aggravated this problem, leading to higher binding ratios.

**Figure 4:**
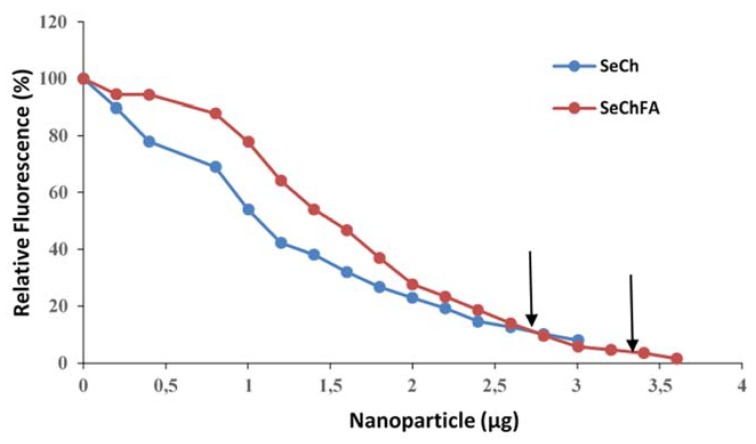
Ethidium bromide intercalation assay showing siRNA compaction of SeCH and SeChFA: Arrows indicate end-points.

Ethidium bromide intercalation was used to investigate the degree of compaction of the siRNA by the nanoparticles and differs in its mechanism from that of the gel retardation assay. Fluorescence dropped steadily as the nanoparticles displaced the intercalated ethidium bromide. The targeted nanoparticles showed higher endpoints than the nontargeted nanoparticles (Figure 4), with a similarity in the 2.5–3 µg range. Although, these results are not exactly consistent with that seen for the gel retardation assay (Figure 3), they provide a useful indication for the complete complexation of the siRNA. Furthermore, the measurement of fluorescence is a more sensitive assessment compared to the simple visualization of fluorescence, as evidenced by the lower binding ratios seen for the ethidium bromide intercalation assay compared to the gel retardation assay.

**Figure 5:**
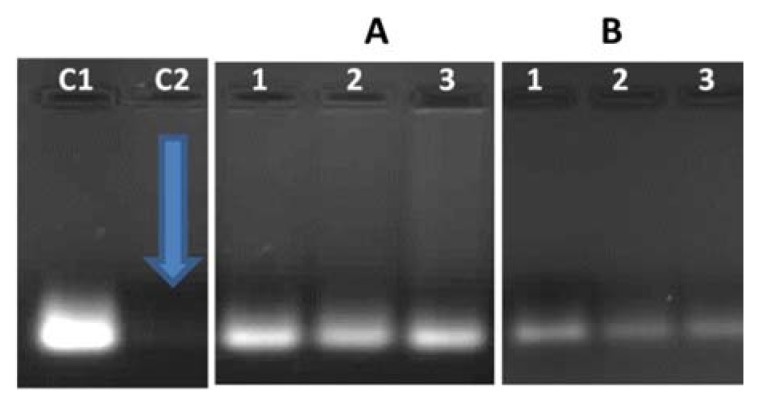
Nuclease digestion studies of NP-siRNA nanocomplexes. C1: siRNA only (0.5 µg/mL); C2: siRNA treated with RNase A; gel (**A**) lanes 1–3: siRNA-SeCh at *w*/*w* ratios (1:5, 1:6, and 1:7) and gel (**B)** lanes 1–3: siRNA-SeChFA at *w*/*w* ratios (1:6, 1:8, and 1:10). Gels depict integral bands produced after treatment with SDS.

After incubation in RNase A, RNA integrity was evaluated using gel electrophoresis. The siRNA complexes were intact after incubation, while free siRNA was completely degraded as seen in lane C2 with no band present. Brighter bands were visible with SeCh, indicating better protection using these vectors compared to targeted vectors (Figure 5). This study confirmed the ability of these nanocomplexes to remain stable under simulated physiological conditions.

### 3.3. Cell Viability

**Figure 6:**
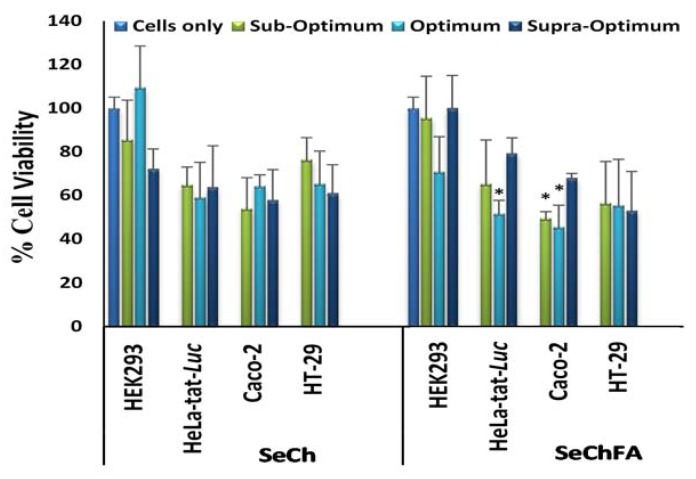
Cell viability studies of nanocomplexes on selected cell lines: Data are represented as mean ± SD (*n* = 3). * *p* < 0.05 vs control. For siRNA-SeCh, *w*/*w* ratios are suboptimum = 1:5, optimum = 1:6, and supra-optimum = 1:7, and for siRNA-SeChFA, *w*/*w* ratios are suboptimum = 1:6, optimum = 1:8, and supra-optimum = 1:10).

In vitro cytotoxicity of the nanocomplexes were evaluated on the Hela-tat-*Luc*, Caco-2, and HT29 cancer cells and the non-cancer HEK293 cells. Results from the MTT assay showed moderate cytotoxicity for most of the nanocomplexes. The highest cytotoxicity (less than 50% cell viability) was observed in the Caco-2 cells with the SeChFA nanocomplex, possibly due to the higher Se concentration required to complex with siRNA. No significant differences in cell viability were observed at different *w*/*w* ratios. The HEK293 cells seemed to tolerate the nanocomplexes best and also showed selective toxicity of the nanocomplexes (Figure 6).

### 3.4. Gene Silencing

**Figure 7:**
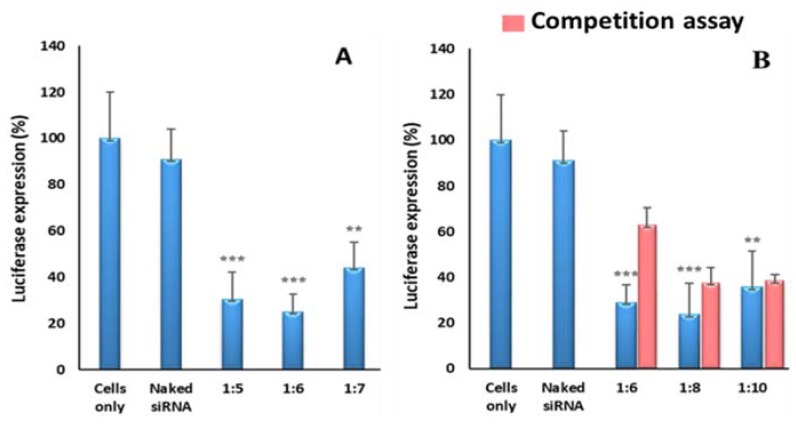
Luciferase gene silencing using (**A**) SeCh and (**B**) SeChFA nanocomplexes in Hela-tat-*Luc* cells at suboptimum, optimum, and supra-optimum *w*/*w* ratios: Data are represented as mean ± SD (*n* = 3). *** *p* < 0.001 and ** *p* < 0.01 vs control.

Nanoparticle transfection efficiency was assessed in the Hela-tat-*Luc* cells stably expressing the luciferase gene. Transfection efficiency was evaluated through measurement of *Luc* gene silencing using synthesised nanocomplexes at different *w*/*w* ratios. The SeChFA nanocomplex showed a marginally higher gene silencing that the nontargeted SeCh complex (Figure 7). However, all nanocomplexes (targeted and nontargeted) showed significantly higher gene silencing efficiency compared to naked siRNA, which did not evoke significant gene silencing, reducing the luciferase expression by only 9%. There were no significant differences in knockdown efficiency between targeted and nontargeted nanocomplexes, indicating that nonspecific endocytosis did play a role in cellular uptake.

The competition assay was performed to examine if the folate receptor played a significant role in endocytosis. Folate receptors were initially blocked with an excess of folic acid prior to addition of nanocomplexes. Gene silencing decreased across all ratios of the targeted nanocomplexes, however not significantly (Figure 7). The suboptimum ratios reflected the lowest silencing capacity compared to the other ratios. This result indicated that both nonspecific endocytosis and folate receptor endocytosis played a role in the uptake of the nanocomplexes and confirmed that the targeted nanocomplexes entered the cell membrane to some degree through folate-receptor mediated endocytosis.

### 3.5. Molecular Docking Studies

**Figure 8:**
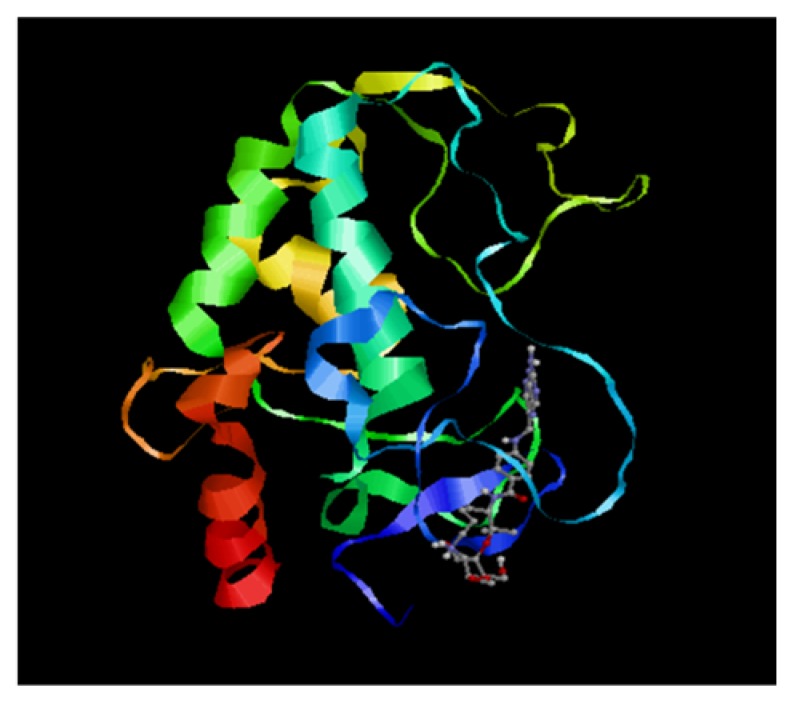
Receptor-ligand interactions of SeChFA with the folate receptor, FOLR1. The green, turquoise, red, yellow and blue ribbons represent the the alpha and beta sheets of the respective proteins within the molecular structure of the folate receptor. The targeted nanoparticle can be seen interacting with the active site residues (blue).

Docking studies on the folate receptor, a glycosylphosphatidylinositol surface receptor, was investigated using the targeted nanoparticles. SeChFA had a binding energy of −4.4 kcal mol^−1^, which according to a previous report suggests lower activity since this binding energy was >−6.0 kcal mol^-1^ [41]. However, that particular study examined binding to traverse the blood brain barrier, a difficult barrier to cross, while the current study focused on uptake into cervical cancer cells in vitro. The interaction of SeChFA was mediated by one active site residue, Lys46 (Figure 8). Overall, the energy level seemed sufficient to facilitate receptor-mediated uptake as evidenced by the competition assay (Figure 7), although this was not the only route of uptake.

### 3.6. Selenium Uptake

**Figure 9:**
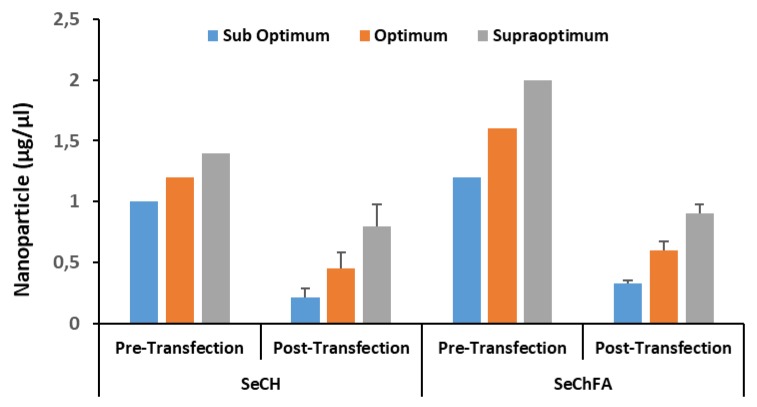
The concentration of selenium determined by ICP-OES before (pre−) and after (post−) transfection in HeLa-tat*-Luc* cells.

We examined the quantitative cellular uptake of SeNPs using ICP-OES. Hela-tat-Luc cells were transfected with folate targeted SeChFA and nontargeted SeCh complexes. SeNP uptake was dose-dependent and higher in the targeted SeChFA. Intracellular concentrations were reflective of the *w*/*w* ratios used in RNA complexing. The amount of Se in the cell was lower than the initial amount used in transfection for all ratios (Figure 9). Despite the somewhat higher uptake with targeted nanoparticles, our results showed no significant differences compared to nontargeted SeNPs. This was consistent with our transfection and competition studies which suggest nonspecific endocytosis played a significant role in the uptake of both targeted and nontargeted vectors.

## 4. Discussion

Se has been studied over the years for its potential chemopreventive, antioxidant, and anticancer activities. However, its use as a delivery nanoparticle is yet to be thoroughly investigated and understood. In this study, we formulated colloidal monodisperse SeNPs with a chitosan shell for stability and to bind the anionic siRNA. The use of a chitosan shell and selenium core was previously utilized for the delivery of F*Luc*-mRNA and showed significant transgene expression and low cytotoxicity in vitro [28]. The choice of this particular molecular weight of chitosan used was based on studies conducted earlier in out laboratory [28,42,43,44,45]. This inorganic SeNP core and a cationic chitosan shell were further modified with a targeting folic acid moiety for cancer cell-specific delivery. Chitosan has previously been used to alter the stability and morphology of inorganic nanoparticles in numerous studies due to its suitability in biomedical applications owing to its non-immunogenicity and biodegradability in vivo [16,44,45,46,47]. Furthermore, it promotes nanoparticle retention in the cell, thus improving its bioactivity. The concentration (0.1%) used in this study has been reported to form stable nanoparticles with high homogeneity [27]. Although chitosan can form nanoparticles on their own, the addition of selenium as a core was strategic to derive the important micronutrient and anticancer properties of selenium [25] and the biocompatible and cationic properties of chitosan for binding of siRNA. Hence, this acquired synergism was hoped to favourably impact the nanoparticle’s biological activity. The use of folate as a ligand has been widely reported due to its affinity for folate receptors, especially on cancer cells [43,48,49,50]. The folate receptor is overexpressed in most cancers and has been used in developing targeted therapies [51].

The nanoparticle and nanocomplex sizes were all below 100 nm, which was ideal for delivery studies and passive or active tumour targeting since nanoparticles greater than 200 nm are known to be readily scavenged in vivo by the reticuloendothelial system effect [52]. Particle size and ζ potential strongly influence transfection efficiency. Stability of nanocomplexes in an aqueous medium, as determined from their ζ potential, is important in determining and assessing their future biomedical application, especially in drug and gene delivery. A ζ-potential <−25 mV and >+25 mV is considered colloidally stable [53]. Based on this, the SeCh nanocomplex was more stable than the targeted nanocomplex. SeNPs, as expected, did not possess the necessary nanocarrier properties and hence needed functionalization. The ζ-potential also represents the electrostatic ionic charge at the surface of a nanoparticle. Positively charged particles, as produced in this study, are important for nucleic acid binding and accumulate at the negatively charged cell membrane facilitating cellular uptake [54]. Complexing of siRNA to cationic nanoparticles was through ionic interactions with subsequent neutralization of negative charges on siRNA as evidenced by zeta potential measurements forming overall positively charged particles. Complexing siRNA through ionic interactions is favoured as it preserves biological activity of the nucleic acid attached [8] and facilitates entry through the negatively charged cell membrane via specific and nonspecific means.

The nanoparticles further showed the ability to protect the siRNA cargo from RNase A degradation. This assay mimics in vivo conditions to ascertain the integrity of the siRNA after entry into the cell and to determine whether the gene of interest would be silenced. Nanocomplex cytotoxicity in vitro is essential in determining its future use as a delivery vector in a clinical setting. Minimal cytotoxicity was observed in the noncancer HEK293 cells compared to the other three cancer cell lines. Although, the HeLa-derived KB cells have often been used as a folate receptor model, HeLa cells themselves have also been used due to their high expression of the folate receptor as well. Hence, since this study examined luciferase gene silencing, it was integral to use a stably transformed cell line such as the HeLa-tat-*Luc* which has been reported previously [50,55]. In these HeLa-tat-*Luc* cells, only the SeChFA nanocomplexes showed cell viability below 60% at the optimum binding ratio. This seemed to mirror the luciferase gene silencing results with most significant gene silencing observed for these complexes at the optimum ratio. Gene silencing efficiency was slightly better for the targeted nanocomplexes than the nontargeted nanocomplexes. This small difference between targeted and nontargeted nanocomplexes has been reported previously in similar studies [2]. The folate receptor competition assay did confirm receptor-mediated uptake of the SeChFA nanocomplexes. Folate-receptor mediated endocytosis played a small role as evidenced by the slightly better gene silencing and cellular uptake of targeted nanoparticles. It is plausible that passive targeting also played an important role in the uptake of the cationic nanoparticles, even without folate targeting. Furthermore, the ICP cellular uptake studies provided evidence that the Se accumulation in the cell was lower than the initial amounts used in transfection for all ratios, suggesting lower uptake and internalization of the SeNPs. This was not unexpected as previous studies have reported chitosan functionalised SeNPs accumulating in culture medium with amounts as low as 1% in the cell substantially altering cell function [56]. Despite the somewhat higher uptake with targeted nanoparticles, our results showed no significant differences compared to nontargeted SeNPs. This is consistent with our transfection and competition studies which suggest nonspecific endocytosis played a major role in uptake of both targeted and nontargeted nanocomplexes.

Investigations on the interactions of folate-targeted nanoparticles with the receptor FOLR1 through molecular docking studies further corroborated our gene silencing results showing interaction of SeChFA with the receptor active site, although not at the highest level. This was expected as the addition of Se to the nanocarrier system could have interfered with ligand–receptor interactions. Hence, further understanding of the uptake mechanisms involved during transfection is crucial in designing an optimized delivery vehicle that is safe, stable, and able to silence a gene of interest successfully. Future efforts would be to identify and concentrate on the silencing of a specific disease or cancer.

## 5. Conclusions

These Se-based nanocarriers exhibited the desirable properties required for a delivery vehicle. They were spherical, were nanoscale in size, have moderate to good colloidal stability, and have shown the ability to complex and compact the anti-*Luc*-siRNA. Both nanocarriers adequately protected the siRNA from RNAse A degradation. Minimal toxicity was observed in the cells studied, with cancer cell-specific selectivity of the nanocomplexes being demonstrated thorough low cytotoxicity in the non-cancer cell line, HEK293. Gene silencing investigated in the modified Hela-tat-*Luc* cells showed significant luciferase gene silencing by both nanocomplexes compared to that of naked siRNA, which we attribute to reduced uptake and degradation of the naked siRNA by RNases. We have further provided evidence that the SeChFA nanocomplexes were able to enter the cells via the folate receptors overexpressed on the HeLa cell surface. This study opens a new avenue for the synergistic treatment of cancer, combining selenium’s bioactivity (as described earlier) with that of the therapeutic cargo, and holds promise for the future of SeNP-mediated gene silencing.

## Figures and Tables

**Table 1:** Size and ζ potential of nanoparticles and nanocomplexes.

Nanoparticle	Size (nm)	ζ Potential (mV)	End-Point Ratios (Optimum Binding)		
			*w*/*w*	*Size*	*ζ Potential*
**SeNP**	85.3 ± 8	−14.8 ± −3.6	n/a	n/a	n/a
**SeCh**	59.6 ± 0.1	21.0 ± 0.2	1:6	57.1 ± 0.3	31.8 ± 1.2
**SeChFA**	75.6 ± 1.4	9.0 ± 0.3	1:8	63.3 ± 0.7	19.3 ± 0.8

n/a: It means not applicable to these NPs. I have changed it to n/a for better clarity.

## Supplementary Materials

The following are available online at https://www.mdpi.com/2227-9059/8/4/76/s1.
