# Supplementary material for: Polymerized Selenium Nanoparticles for Folate-Receptor-Targeted Delivery of Anti-Luc-siRNA: Potential for Gene Silencing
Source: Biomedicines. 2020 Apr 5;8(4):76. doi: 10.3390/biomedicines8040076 (PMC7235796; doi:10.3390/biomedicines8040076)
Supplement: Supplementary file 1 [file biomedicines-08-00076-s001.pdf]

Supplementary Material

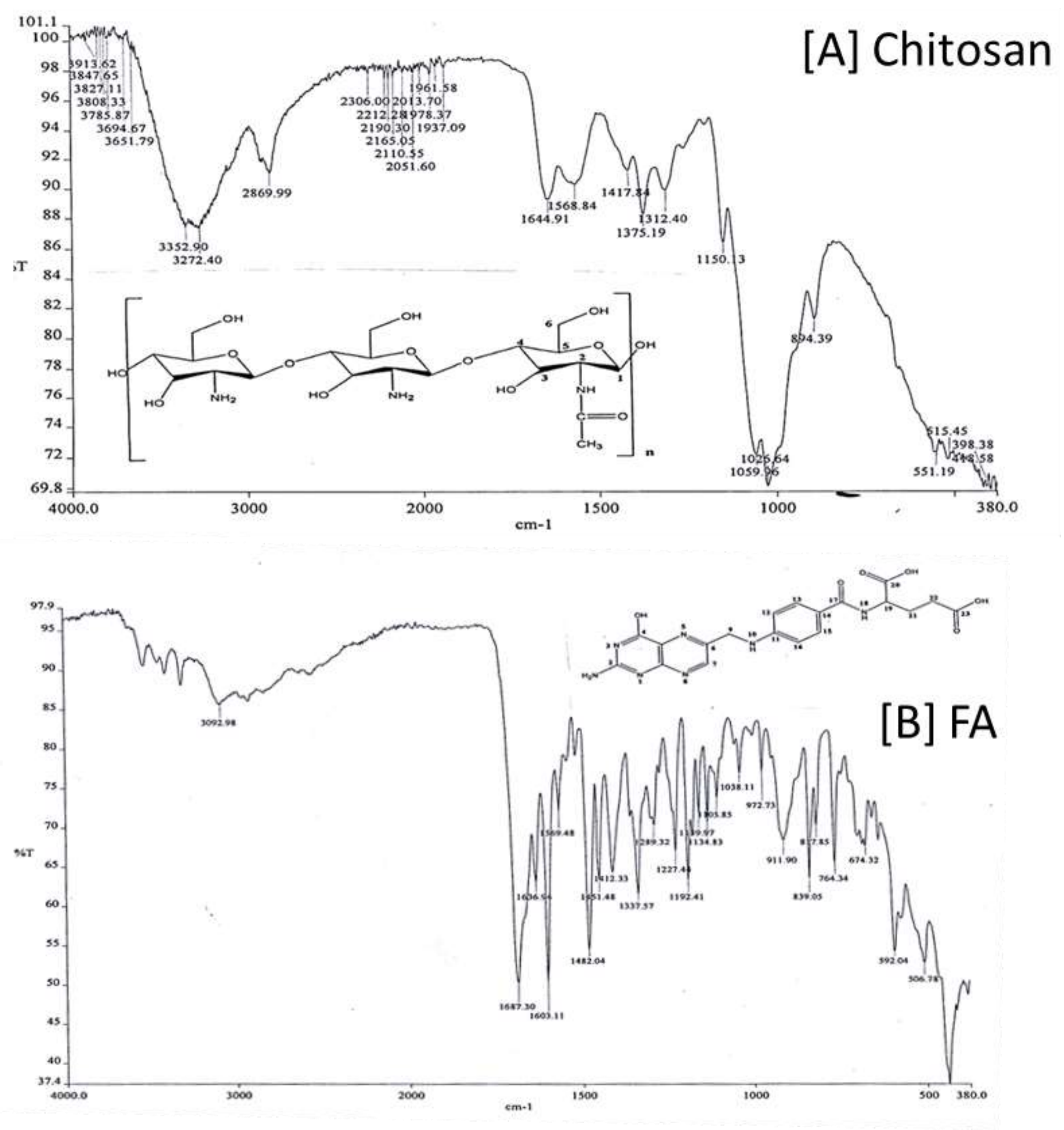

Figure S1: FTIR of (A) Chitosan (Ch), and (B) Folic acid (FA).

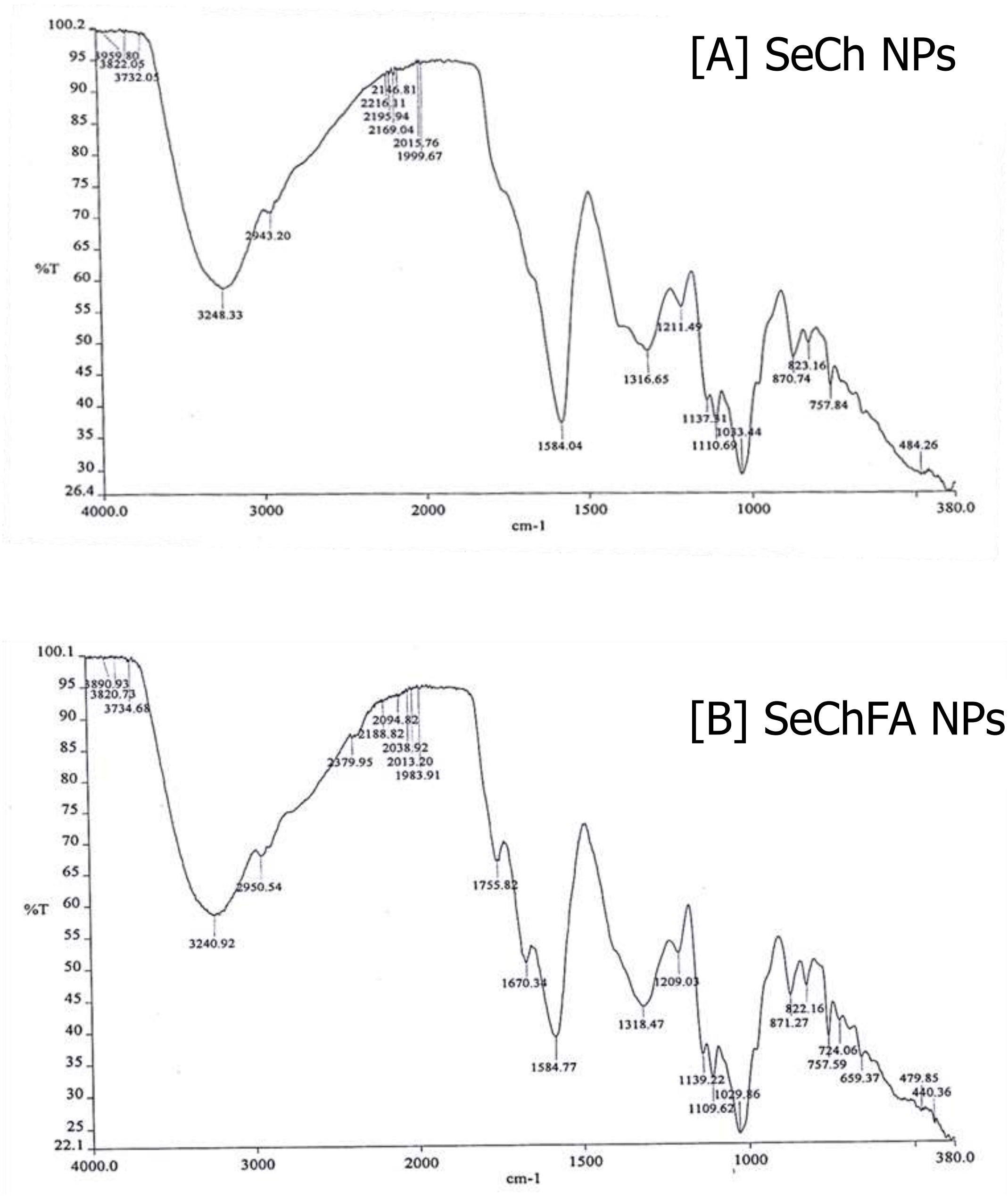

Figure S2: FTIR of (A) SeCh NPs and (B) SeChFA NPs.
